# Progesterone Attenuates Microglial-Driven Retinal Degeneration and Stimulates Protective Fractalkine-CX3CR1 Signaling

**DOI:** 10.1371/journal.pone.0165197

**Published:** 2016-11-04

**Authors:** Sarah L. Roche, Alice C. Wyse-Jackson, Violeta Gómez-Vicente, Pedro Lax, Ana M. Ruiz-Lopez, Ashleigh M. Byrne, Nicolás Cuenca, Thomas G. Cotter

**Affiliations:** 1 Cell Development and Disease Laboratory, Biochemistry Department, Biosciences Institute, University College Cork, Cork, Ireland; 2 Departamento de Óptica, Farmacología y Anatomía, Universidad de Alicante, Alicante, Spain; 3 Departamento de Fisiología, Genética y Microbiología, Universidad de Alicante, Alicante, Spain; University of Michigan, UNITED STATES

## Abstract

Retinitis pigmentosa (RP) is a degenerative disease leading to photoreceptor cell loss. Mouse models of RP, such as the rd10 mouse (B6.CXBl-Pde6brd10/J), have enhanced our understanding of the disease, allowing for development of potential therapeutics. In 2011, our group first demonstrated that the synthetic progesterone analogue ‘Norgestrel’ is neuroprotective in two mouse models of retinal degeneration, including the rd10 mouse. We have since elucidated several mechanisms by which Norgestrel protects stressed photoreceptors, such as upregulating growth factors. This study consequently aimed to further characterize Norgestrel’s neuroprotective effects. Specifically, we sought to investigate the role that microglia might play; for microglial-derived inflammation has been shown to potentiate neurodegeneration. Dams of post-natal day (P) 10 rd10 pups were given a Norgestrel-supplemented diet (80mg/kg). Upon weaning, pups remained on Norgestrel. Tissue was harvested from P15-P50 rd10 mice on control or Norgestrel-supplemented diet. Norgestrel-diet administration provided significant retinal protection out to P40 in rd10 mice. Alterations in microglial activity coincided with significant protection, implicating microglial changes in Norgestrel-induced neuroprotection. Utilizing primary cultures of retinal microglia and 661W photoreceptor-like cells, we show that rd10 microglia drive neuronal cell death. We reveal a novel role of Norgestrel, acting directly on microglia to reduce pro-inflammatory activation and prevent neuronal cell death. Norgestrel effectively suppresses cytokine, chemokine and danger-associated molecular pattern molecule (DAMP) expression in the rd10 retina. Remarkably, Norgestrel upregulates fractalkine-CX3CR1 signaling 1 000-fold at the RNA level, in the rd10 mouse. Fractalkine-CX3CR1 signaling has been shown to protect neurons by regulating retinal microglial activation and migration. Ultimately, these results present Norgestrel as a promising treatment for RP, with dual actions as a neuroprotective and anti-inflammatory agent in the retina.

## Introduction

Retinitis pigmentosa (RP) encompasses a set of hereditary diseases resulting in a progressive loss of rod and subsequently cone photoreceptors, leading to eventual blindness [1]. The rd10 mouse model of RP possesses a mutation in the *phosphodiesterase-6b* (*pde6b)* gene [2–4]. A suitable model for studying cell death in RP, the course of photoreceptor cell loss and subsequent retinal degeneration in this mouse closely resembles disease progression in humans [5–9]. In 2011, our group showed that the synthetic progestin ‘Norgestrel’ works as a neuroprotective agent in the retina, thus identifying it as a potential treatment for RP [10]. Published studies have since shown that Norgestrel, working through progesterone receptors [11], significantly increases production of basic fibroblast growth factor (bFGF) and leukemia inhibitory factor (LIF) in the retina [10, 12]. These growth factors likely act directly on photoreceptors to provide neuroprotection, through an upregulation of pro-survival and downregulation of apoptotic pathways [13].

We are beginning to understand more about the mechanism of action of Norgestrel on photoreceptors. However, the effect of Norgestrel on other cell types in the retina has yet to be studied in detail. Particularly, can Norgestrel work on the resident retinal macrophages, the microglia? Macrophages are essential in the clearance of cell debris, maintaining homeostasis and facilitating tissue repair following injury in the central nervous system [14–16]. However, macrophages have also been implicated in the pathology of many neurodegenerative diseases, in which neuroinflammation is considered to be a hallmark [17]. Previous studies have highlighted a detrimental role for microglia as drivers of retinal cell degeneration [18–21]. In the rd10 mouse, microglia respond to the mutation as early as P5 and are found in close association with the photoreceptors during the period of initial cell loss [9]. Other studies have shown that when microglial cells are either genetically ablated, their phagocytic ability inhibited [18], or their pro-inflammatory activities dampened by anti-inflammatory drug administration [19], disease progression is attenuated in the rd10 retina.

The aim of this study was to develop our understanding of how Norgestrel works to provide neuroprotection in a mouse model of RP, with a particular focus on the response of retinal microglia to Norgestrel, if any. Interestingly, neurosteroid signaling has previously been shown to dampen damaging inflammatory gene expression [22–25]. We examined interactions between rd10 retinal microglia and the 661W photoreceptor-like cell line *in vitro* and show that microglial-driven 661W cell death is abrogated by pre-treating microglia with Norgestrel. This result highlights a novel role for Norgestrel as an anti-inflammatory agent, working directly on microglia to reduce their pro-inflammatory phenotype and consequentially promote survival of photoreceptors. We also provide evidence for Norgestrel upregulating the neuroprotective compound fractalkine in the rd10 retina, which could further dampen microglial activity. In support of this hypothesis, treatment of primary rd10 microglia with recombinant fractalkine reduces their ability to kill 661W cells. Future studies will be aimed at further investigating the role that fractalkine plays in Norgestrel’s neuroprotective effects.

## Materials and Methods

### Mice

All animals were handled and maintained following the Association for Research in Vision and Ophthalmology statement for the Use of Animals in Ophthalmic and Vision Research (License Number AE19130/P009). Experiments were approved by University College Cork Animal Experimentation Ethics Committee and were performed using both male and female homozygous rd10/rd10 mice (B6.CXBI-Pde6b^rd10^/J) and C57 mice (C57BL/6J). Mice were supplied by the Biological Services Unit, University College Cork and were humanely euthanized by cervical dislocation.

### Norgestrel-supplemented diet

Norgestrel-supplemented and control diets were manufactured by Testdiet (Middlesex, UK). Norgestrel was added to the chow at a concentration of 0.05% (500ppm). Dams of rd10 pups were given a Norgestrel-supplemented diet (LabDiet 5053, custom diet containing D (-) Norgestrel (N2260) (Sigma Aldrich, Missouri, US) when the pups were P10 to allow pups to receive Norgestrel in the milk. This equates to a daily intake of approximately 80mg/kg, assuming a 30g mouse consumes around 5g of food/day. Specialized diet soaked in water was also added to the cage in order for pups to receive Norgestrel directly, as soon as they began to eat. These pups were also given Norgestrel-supplemented diet post-weaning. With control litters, diet changes were replicated with a control diet (LabDiet 5053 control diet). Dams were given regular chow once pups were weaned.

### Immunohistochemistry on retinal sections

Eyes were enucleated and fixed at room temperature in 4% paraformaldehyde (PFA) for 1.5 h. Following washes, eyes were cryo-protected in 15% sucrose in 1xPBS for 1 h, 20% sucrose for 1.5 h and 30% sucrose overnight, all at 4°C. Eyes were submerged and frozen in cryochrome (Thermo Scientific, Waltham, US) and sectioned on a cryostat (Leica, Wetzlar, Germany). Sections (7μm) were collected on Superfrost glass slides (Fisher Scientific, Waltham, US) and stored at -80°C. Sections were blocked and permeabilized with 0.1% Triton X and 5% donkey serum in 1xPBS for 30 min and incubated with primary antibody diluted in 5% donkey serum overnight at 4°C. Table 1 lists the details of all primary antibodies used. Following washes, sections were incubated with secondary antibody (Alexa Fluor donkey anti-mouse/rabbit/goat with either a 488 or 594 fluorescent probe; Molecular Probes &/or FITC–PNA (Vector Labs, Burlingame, US.)) with the nuclear stain, Hoechst (Thermo Scientific) for 1 h at room temperature. Eliminating the primary antibody in solution served as a negative control. Sections were mounted using Mowiol (Sigma) with anti-fade agent Dabco (Sigma). Images were taken from central and peripheral regions of the retina. Regions identified as central and peripheral are shown in S1 Fig.

**Table 1:** List of antibodies and lectins used in western blotting and immunofluorescence.

Antibody/Lectin	Supplier	Catalogue #	Host	Dilution Factor
**Rhodopsin**	Millipore	AB9279	Rabbit poly	IF 1:200
**FITC-PNA**	Vector Labs	FL-1071	-	IF 1:500
**Iba1**	Wako	019–19741	Rabbit poly	IF 1:500
**Iba1**	Novus Bio	NB100-1028	Goat poly	IF 1:400
**CD68**	AbD Serotec	MCA1957GA	Rat mono	IF 1:500
**iNos**	Cell Signalling	13120	Rabbit mono	IF 1:200
**MRC1**	Abcam	AB64693	Rabbit poly	IF 1:1000
**Cone Arrestin**	Millipore	AB15282	Rabbit poly	IF 1:1000
**PR A/B**	Abcam	AB2764	Mouse mono	IF 1:100
**PGRMC1**	Sigma	HPA002877	Rabbit poly	IF 1:100
**PGRMC2**	Santa Cruz	sc-104609	Goat poly	IF 1:200
**mPRα**	Santa Cruz	sc-50111	Goat poly	IF 1:100
**mPRβ**	Abcam	ab46534	Rabbit poly	IF 1:100
**mPRγ**	Abcam	ab79517	Rabbit poly	IF 1:100
**GFAP**	Sigma	G3893	Mouse mono	IF 1:300
**Glutamine Syn.**	Millipore	MAB302	Mouse mono	IF 1:100
**NFM**	Abcam	ab7794	Mouse mono	IF 1:500
**Syntaxin 1**	Millipore	AB5820	Rabbit poly	IF 1:500
**Calbindin**	Swant	CB-38a	Rabbit mono	IF 1:500
**CHX10**	Santa Cruz	sc-21690	Goat poly	IF 1:100
**Fractalkine**	Abcam	ab25088	Rabbit poly	IF 1:1000 WB 1:1000

### Immunohistochemistry on cells

Microglia and 661W cells cultured on polylysine-coated coverslips in 24 well plates were fixed with 4% PFA for 10 min at room temperature. Cells were blocked and permeabilized in 0.1% Triton X and 5% donkey serum in 1xPBS for 10 min and incubated with primary antibody diluted in 5% donkey serum overnight at 4°C. Table 1 lists the details of all primary antibodies used. Following washes, coverslips were incubated with secondary antibody (Alexa Fluor donkey anti-mouse/rabbit/goat with either a 488 or 594 fluorescent probe) with the nuclear stain, Hoechst (Thermo Scientific) for 1 h at room temperature. Eliminating the primary antibody in solution served as a negative control. Coverslips were mounted on to glass slides using Mowiol.

### Microscopy

Retinal sections and cell preparations were viewed using a Leica DM LB2 microscope with Nikon Digital Sight DS-U2 camera, using 10x, 40x and 100x objectives. Images were taken using the software NIS-Elements version 3.0, Nikon, Japan. Immunofluorescence on retinal sections was performed on at least three mice of each group, at each time-point. Immunofluorescence on cell preparations was also performed in triplicate. Confocal micrographs were taken using an Olympus Fluoview FV1000 laser scanning confocal microscope, using 20x and 60x objectives. Images were taken using the software Olympus Fluoview Ver 4.1a and are represented as single slices in the XY plane or maximum intensity projections from acquisition of z-stacks. Identical microscope settings were used when visualizing specific markers across all timepoints and treatments.

### Terminal dUTP nick-end labeling (TUNEL) of fragmented DNA

DNA strand breaks in retinal cell nuclei and 661W cells were detected by terminal dUTP nick-end labeling (TUNEL) on fixed tissue and cells. Frozen retinal sections or coverslips with cells were permeabilzed with 0.1% Triton X for 2 min followed by incubation with terminal deoxynucleotidyl transferase (Promega, Wisconsin, US) and fluorescein-12-dUTP (Roche, Risch-Rotkreuz, Switzerland) according to manufacturer’s instructions. Nuclei were counterstained with Hoechst 33342 (1μg/mL) (Sigma). Sections and cells were incubated at 37°C for 1 h in a humidified chamber and following several washes in 1xPBS, were mounted in Mowiol. Sections were viewed under a fluorescence microscope (Leica DM LB2). In co-culture assays, TUNEL-positive 661W cells could easily be distinguished from microglia based on nuclear size and phase contrast of cell preparations. For each treatment on mice, at least three animals were used and three fields (x40 magnification) per section of at least three different sections were evaluated. For each treatment on coverslips, 6 coverslips were used per treatment and 50–200 661W cells analyzed per coverslip for TUNEL positivity.

### ERG recordings

Dark-adapted (12 h) mice were anaesthetized with an intraperitoneal injection of ketamine (100 mg/kg)—xylazine (4 mg/kg) and maintained on a thermal blanket at 38°C for the entire procedure. 1% tropicamide (Alcon Cusí, Barcelona, Spain) was applied topically on the eyes to dilate the pupils and viscotears (Novartis, Barcelona, Spain) was instilled on the corneas to prevent dehydration and optimize contact with the recording electrodes (DTL fiber with an X-Static silver-coated nylon conductive strand; Sauquoit Industries, Scranton, PA). The reference electrode was a 25-gauge platinum needle inserted under the scalp and a gold electrode, located in the mouth, acted as ground. Animal handling and preparation was done under dim red light and the mice were placed on a Faraday cage where all the recordings were performed in total darkness. Scotopic flash-induced ERG responses were recorded simultaneously from both eyes in response to light stimuli produced with a Ganzfeld illuminator. Light stimuli of 10 ms of duration were presented at 11 different increasing luminances, ranging from −5.2 to 0 log cd s/m2. Three to ten consecutive recordings were averaged for each light presentation. The interval between stimuli was 10 s for dim flashes (−5.2 to −1.4 log cd s/m2) and up to 20 s for the highest luminance’s (−0.8 to 0 log cd s/m2). ERG signals were amplified and band-pass filtered (1–1000 Hz, without notch filtering) using a DAM50 data acquisition board (World Precision Instruments, Aston, UK). Stimuli presentation and data acquisition (4 kHz) were performed using a PowerLab system (AD Instruments, Oxfordshire, UK). Recordings were analyzed off-line. The amplitude of the a-wave was measured from the baseline and the amplitude of the b-wave was measured from the trough of the a-wave to the peak of the b-wave.

### Culture of cell lines

Experiments were carried out using the mouse 661W cone photoreceptor-derived cell line, generously provided by Dr Muayyad Al-Ubaidi (Department of Cell Biology, University of Oklahoma, Health Sciences Centre, Oklahoma City, OK, USA). This cell line was previously validated by this group [11] through real-time quantitative polymerase chain reaction (rt-qPCR) analysis for cone specific opsins; blue cone opsin (Opn1sw) and red/green opsin (Opn1mw). Cells were cultured in Dulbecco’s Modified Eagle’s medium (DMEM) (Sigma) supplemented with 10% fetal calf serum (FCS) and 1% penicillin streptomycin (PS) and maintained at 37°C in a humidified 5% CO_2_ atmosphere. To analyze the effects of Norgestrel (Sigma) on 661W cells, cells were seeded in to the appropriate culture vessel and allowed to attach overnight. Cells were then washed three times with warmed 1xPBS and complete or serum-free medium supplemented with 20 μM Norgestrel or DMSO vehicle (Sigma) added. After incubation for the indicated times, cells were detached using accutase (Sigma) and, together with their supernatant, centrifuged and washed with 1x PBS to leave a whole cell pellet.

### Western Blotting

Whole retinas were lysed in RIPA buffer (Thermo) containing protease inhibitors (Thermo). Lysates were centrifuged at 10,000 rpm at 4°C for 30 min. Supernatant was stored at -80°C. Protein concentration was measured using a Bradford assay (Bio-Rad, Hercules, US). 4–15% gradient gels (Bio-Rad) were used for SDS-PAGE and proteins were then transferred to a nitrocellulose membrane. 30μg of protein was loaded per sample. Total protein levels are an accurate way of verifying equal loading [26] and so were analyzed using REVERT total protein stain (LiCor, Lincoln, US) as per manufacturer’s instructions and imaged on a LiCor scanner in the 700 channel. Membranes were blocked using Odyssey blocking buffer (LiCor) for 30 min and probed with fractalkine primary antibody (Abcam, Cambridge, UK) in blocking buffer and 0.1% Tween 20 for 4 nights at 4°C. Membranes were washed using TBS-T and probed with rabbit 800 secondary antibody (LiCor). Membranes were washed using TBS and imaged on a LiCor scanner.

### Nitrite parameter assay

Cell culture media from rd10 primary microglia treated with 20 μM Norgestrel or DMSO over 24 hr was collected. Media was centrifuged at 1000 rpm for 5 min to remove particulates. Supernatant was stored at -20°C. Samples were analyzed for endogenous nitrite (R&D systems, Minneapolis, US) according to manufacturer’s instructions.

### Culture of primary microglial cells

A protocol for isolating and culturing retinal microglia was adapted from a previously published protocol [27]. Briefly, retinas were dissected from the eyes of P16 rd10 mice, ensuring minimum contamination with vitreous body and retinal pigment epithelium. Four retinas were pooled, cut into small pieces and incubated for 40 min at 37°C in 1 ml 1xPBS with 1 mg/ml collagenase type I (Sigma), 0.3 mg/ml DNase I (Roche, Basel, Switzerland), and 0.2 mg/ml hyaluronidase (Sigma). The cell suspension was filtered through a 70-μm cell strainer (Becton Dickinson, Franklin Lakes, US). Cells were washed twice with 10 ml DMEM/10% FCS/1% PS and resuspended in 15ml DMEM/10% FCS/1% PS. To isolate the mononuclear cells, the suspension was gently added to 15ml Ficoll paque premium reagent (GE Life Sciences, Buckinghamshire, UK) and centrifuged for 20 min at 2000 rpm without the brake in a Beckmann GS-6R centrifuge. The interphase was removed carefully and washed twice with 10 ml DMEM/10% FCS/1% PS. The cells were cultured for 3 days, on either polylysine-coated coverslips in 24 well plates or T25 cm^2^ flasks containing DMEM/10% FCS/1% PS. Culture purity was confirmed by the absence of retinal astrocytes, Müller glia, rods, retinal ganglion, amacrine, horizontal and bipolar cells, as assessed by immunofluorescence (S2 Fig). Although rare, some debris from these cell types was found in culture (S2A Fig, inserts), however all Hoechst-positive cells stained positively for ionized calcium-binding adaptor molecule 1 (Iba1) and so were confirmed to be microglia (S2B Fig). P16 was chosen as the time-point to harvest primary cells. Microglia respond and migrate to the mutated photoreceptors becoming activated by P15 in the rd10 retina compared to C57 control [9]. This time-point therefore represents a time when microglia have responded to the mutated photoreceptors but photoreceptor cell death has not yet taken place. This eliminates the response of microglia to accumulating photoreceptor cell debris.

### Culture of 661W cells in microglial-conditioned media

A total of six retinas from three P16 rd10 mice were used to make the primary culture for the conditioned media experiment. Primary microglia were cultured in T25cm^2^ flasks for 2 days before treating with Norgestrel or vehicle (DMSO). Cells were exposed to 20 μM Norgestrel in DMSO or vehicle and left for 24 h. Cells were washed with 1xPBS and cultured in fresh DMEM/10% FCS/1% PS for a further 24 h. This conditioned media was collected and added to 661W cells. 661W cells were seeded overnight in 96 well plates (Sarstedt, Numbrecht, Germany) to allow for cell adherence. All cells were then washed three times in 1xPBS and media replaced with either basal microglial media, or conditioned media from treated primary microglial cells (100μl/well). Cells were incubated for 20 h before addition of MTS (3-(4,5-dimethylthiazol-2-yl)-5-(3-carboxymethoxyphenyl)-2-(4-sulfophenyl)-2H-tetrazolium) for a further 4 h.

### Co-culture of rd10 microglia with 661W cells

A total of sixteen retinas from eight P16 rd10 mice were used to make the primary culture for the co-culture experiment with 661W cells following treatment with Norgestrel. Primary microglia were cultured on polylysine-coated coverslips in 24 well plates for 1 day before treating with Norgestrel or vehicle (DMSO). Primary cells were exposed to 20 μM Norgestrel or vehicle and left for 24 h. Cells were washed with 1xPBS prior to the addition of healthy or serum-starved 661W cells that had been treated with Norgestrel or vehicle in the preceeding 3 h. 661W cells suspended in fresh DMEM/10% FCS/1% PS were added to the microglia at a density of 25 x 10^3^ per well. Co-cultures of microglia and 661W cells were left for 24 h before fixation in 4% PFA.

A total of eighteen retinas from nine P16 rd10 mice were used to make the primary culture for the co-culture experiment with 661W cells following treatment with recombinant fractalkine. Primary microglia were cultured on polylysine-coated coverslips in 24 well plates for 1 day before treating with recombinant mouse full-length fractalkine (R&D systems cat. no. 472-FF-025) or vehicle (0.1% BSA in 1xPBS). Primary cells were exposed to 1.2μg/ml fractalkine or vehicle and left for 24 h. Cells were washed with 1xPBS prior to the addition of serum-starved 661W cells. 661W cells suspended in fresh DMEM/10% FCS/1% PS were added to the microglia at a density of 25 x 10^3^ per well. Co-cultures of microglia and 661W cells were left for 24 h before fixation in 4% PFA.

### Quantification from immunohistochemically-labeled sections and cells

Quantification of outer nuclear layer (ONL) thickness and outer segment length in retinal sections was carried out using ImageJ software. Average ONL thickness was measured by taking measurements from at least 20 sections per mouse. Per section, three distinct measurements were taken and averaged. Average outer segment length was measured by taking measurements from at least four sections per mouse. Per section, three distinct measurements of rod or cone outer segment length were taken and averaged. Three mice were analyzed per group. Fluorescence intensity measurements of CD68, iNOS and MRC1 in primary microglia were performed using ImageJ software, by measuring points of intensity within single cells.

### Assessment of cell viability in 661W cells by the MTS assay

Cells seeded overnight (2 x 10^3^ per well 96 well plate) were washed three times in 1xPBS before treatment (conditioned microglial media). After 20 h of treatment incubation, 20μl of MTS solution (Promega) was added to each well and incubated for a further 4 h at 37°C. Viable cells in the presence of phenazine methosulfate (PMS) will reduce the MTS solution to form formazan. Detection and quantification of the formazan crystals was carried out with a microplate reader (Molecular Device Corporation, Sunnyvale, US) at 490nm. 490nm readings taken from non-template wells (media and MTS, without cells) were deducted from actual cellular readings. A further reading at 650nm was taken from all wells and deducted from the 490nm readings to account for any cellular debris. The quantity of formazan product as measured by the amount of 490nm absorbance is directly proportional to the number of living cells in culture. Therefore the absorbance of the formazan formed in ‘control cell wells’ was taken as 100% viability.

### Total RNA Isolation and quantitative real-time polymerase chain reaction (rt-qPCR)

Total RNA was isolated from whole 661W cells and RNA-later (Qiagen, Hilden, Germany) protected tissue using RNeasy Mini Kit (Qiagen), or from primary microglia using RNeasy Micro Kit (Qiagen) following manufacturer’s protocol. Genomic DNA was eliminated in 661W cells/tissue samples using RNase free DNase (Qiagen) and in primary microglia using gDNA eliminator columns (Qiagen) and cDNA was subsequently synthesized using QuantiTect Reverse Transcription Kit (Qiagen). Rt-qPCR was performed using QuantiTect SYBR Green PCR Kit (Qiagen) and 10ng cDNA/well of a 384 well plate (Starstedt AG & Co.) for 661W cells/tissue and 5ng cDNA/well for primary microglia. Plates were run using the Applied Biosystems 7900HT Fast Real-Time PCR System (Life Technologies Ltd., Carlsbad, US) and each set of reactions included both a non-reverse transcription control and a no template sample negative control (data not shown). The protocol consisted of a cycling profile of 30s at 95°C, 60s at 60°C, and 30s at 72°C for 40 cycles. Qiagen QuantiTect Primer assays were used for all genes (Table 2). Melt curve analysis confirmed single PCR product. Relative changes in gene expression were quantified using the comparative Ct (ΔCt, ΔΔCt) method as described by Livak & Schmittgen [28, 29]. The Ct value of the gene of interest was normalized to an average of three endogenous housekeeping genes (Actb, Gapdh and Hprt). This was compared to the normalized control sample–an aged matched control diet rd10 value, representative of all control samples (N = 3) or an equivalent DMSO control (661W cell treatments). Alteration in mRNA expression of genes was defined as fold difference in the expression level in cells/tissue after treatment, relative to that of the control. This is the standard method for presenting rt-qPCR data [28, 29].

**Table 2:** List of Qiagen Quantitect Primer assays used in RT-qPCR.

Gene	Qiagen Primer	Product Size	RefSeq ID#
**Actin**	QT00095242	149	NM_007393
**GAPDH**	QT01658692	144	NM_008084
**HPRT**	QT00166768	168	NM_013556
**PR A/B**	QT00114534	118	NM_008829
**PGRMC1**	QT00250887	131	NM_016783
**PGRMC2**	QT00258608	111	NM_027558
**mPRα**	QT00138145	78	NM_027995
**mPRβ**	QT00123886	67	NM_028829
**mPRγ**	QT01067612	67	NM_028748
**iNOS**	QT01547980	131	XM_001004823
**Arginase**	QT00134288	109	NM_007482
**Mannose Receptor**	QT00103012	132	NM_008625
**HMGB1**	QT00247786	78	NM_010439
**IL-1α**	QT00113505	110	NM_010439
**MIP1Alpha**	QT00248199	107	NM_011337
**MIP1Beta**	QT00154616	110	NM_013652
**MCP1**	QT00167832	118	NM_011333
**MCP3**	QT00171458	117	NM_013654
**Fractalkine**	QT00128345	98	NM_009142
**CX3CR1**	QT00259126	63	NM_009987
**IL-1β**	QT01048355	150	NM_008361
**IL-6**	QT00098875	128	NM_031168
**TNF-α**	QT00104006	112	NM_013693

### Statistical analysis

Values in all graphs represent the mean ± standard error of the mean (SEM) and are representative of at least three individual experiments repeated in triplicate. Data were statistically analyzed using Student t-test and two-way ANOVA (Graph Pad, Prism 6) with values of *p* < 0.05 being considered statistically significant. Values that were more than ± 3 standard deviations from the mean were classed as outliers and removed from analysis. A multi-factor analysis of variance (MANOVA) was used to evaluate the effects of treatment (control vs. Norgestrel-supplemented diet), age and light intensity, on ERG responses. For ERG results, when a 0.05 level of significance was observed, post-hoc pairwise comparisons using Bonferroni's test were carried out.

## Results

### Norgestrel-supplemented diet from P10 results in significant protection of photoreceptors in the rd10 mouse

**Fig 1:**
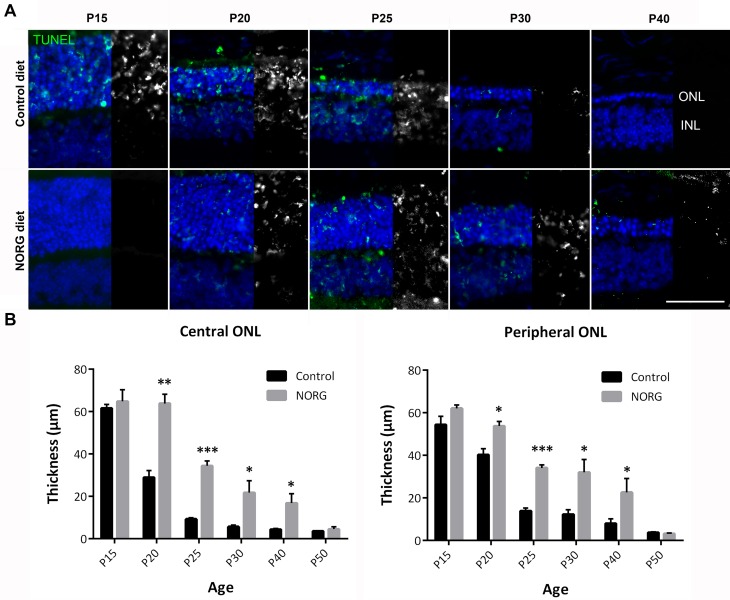
Norgestrel-supplemented diet significantly protects against photoreceptor cell death out to P40 in the rd10 retina. **(A)** Apoptosis of retinal cells was detected by terminal dUTP nick end-labeling (TUNEL; green & greyscale) of DNA strand breaks in cell nuclei. Norgestrel treatment (NORG diet) decreased TUNEL-positive staining in the central P15 and P20 retina compared to control mice (Control diet). N = 3 mice per group. Hoechst (blue) staining reveals the cell layers in the retina. Scale bar 50μm. **(B)** Quantification of outer nuclear layer (ONL) thickness in the central and peripheral retina. N = 3 mice per group, n = 20 sections per mouse. Results are presented as mean ± SEM (t-test, *p<0.05, **p<0.01, ***p<0.005).

Norgestrel has previously been shown to provide significant neuroprotection to the rd10 retina when administered by intra-peritoneal injection from P18 [10]. We sought to evaluate the protection offered by Norgestrel when using a less invasive method of diet-supplementation. We also recognized a need to administer Norgestrel at an earlier age based on recent findings that the rd10 retina displays significant abnormalities prior to photoreceptor cell loss [9]. Dams of newborn rd10s were given a Norgestrel-supplemented diet when their pups reached post-natal day 10 (P10), and all rd10s continued to receive a Norgestrel-supplemented diet post-weaning. Control rd10 litters received an identical diet without Norgestrel. Norgestrel-supplemented rd10’s demonstrated lower levels of TUNEL positivity in the central outer nuclear layer (ONL) at P15 and P20 compared to control (Fig 1A), coinciding with significant preservation of photoreceptors. As expected, control rd10 mice revealed a substantial loss of photoreceptors between P15 and P25 (Fig 1 & S1 Fig) [9]. At P20, ONL thickness in the Norgestrel-fed rd10 mice was comparable to age-matched C57 retina (S1 Fig). Measurement of ONL thickness confirmed significant Norgestrel-induced ONL protection out to P40 in both the central and peripheral retina (Fig 1B). Norgestrel’s neuroprotective effects were lost by P50.

### Norgestrel-supplemented diet results in significant functional preservation in the rd10 retina

**Fig 2:**
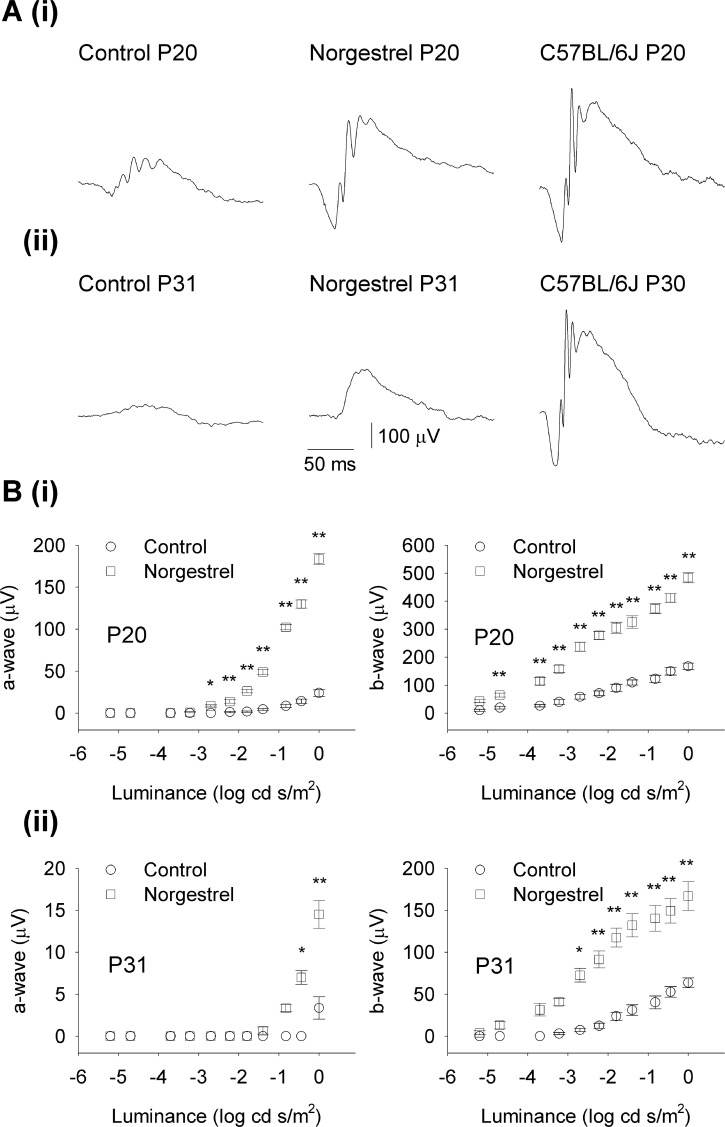
Norgestrel-supplemented diet significantly preserves retinal function in the rd10 retina. **(A)** Representative scotopic ERG waveforms performed on (i) P20 and (ii) P30-31 rd10 mice on a control or Norgestrel-supplemented diet or C57 mice indicating increased neurotransmission in Norgestrel-fed rd10 mice at both time-points. **(B)** Light intensity-response curves of (i) P20 and (ii) P31 rd10 mice on a control (circles) or Norgestrel-supplemented diet (squares), showing statistically significant differences between treatments, in both a- and b-wave amplitudes. N = 4 mice per group, n = 3–10 recordings per mouse. Results are presented as mean ± SEM (MANOVA, Bonferroni post-hoc test, *p<0.05, **p<0.001).

In order to determine functionality of the rd10 retina following Norgestrel-diet supplementation, scotopic electroretinogram (ERG) recordings were taken in mice at P20 and P31, where significant preservation of the ONL was observed (Fig 1). ERG results confirmed functional preservation of the rd10 retina (Fig 2A). As previously described [5, 6], the amplitudes of the a- and b-waves in rd10 mice at P20 were diminished compared to age-matched wild type animals. This decline was significantly attenuated by Norgestrel diet supplementation (Fig 2B). Remarkably, the protective effect of Norgestrel on a- and b-wave amplitudes persisted at P31, where the values corresponding to the b-wave amplitudes were even higher than those of P20 mice fed with control diet.

### Norgestrel-supplemented diet results in significant preservation of photoreceptor outer segments in the rd10 mouse

**Fig 3:**
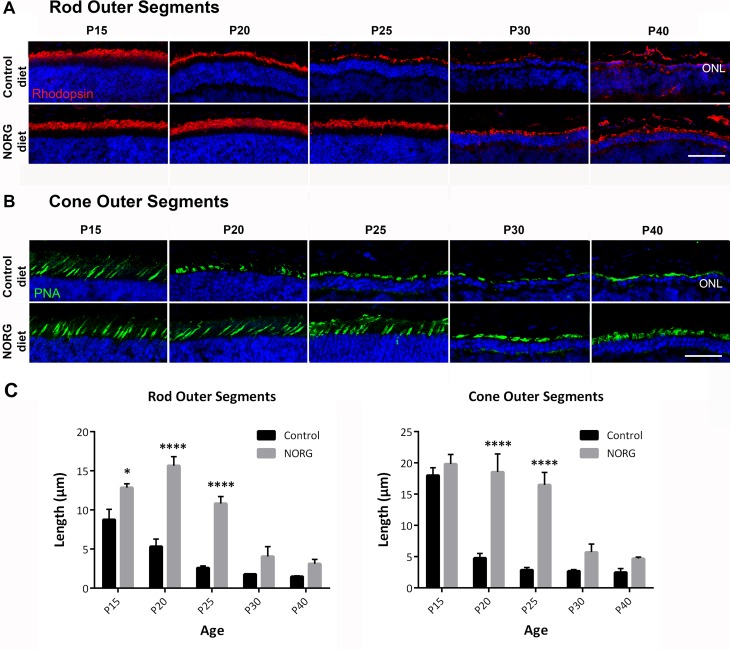
Norgestrel preserves rod and cone outer segment morphology in the rd10 retina. Confocal microscopic images of **(A)** rod (rhodopsin; red) and **(B)** cone outer segments (PNA; green) in the central retina from P15-P40 of control and Norgestrel-fed rd10 mice. Outer segments are shorter and sparser in the control diet retina from P15 for rods (A) and from P20 for cones (B). N = 3 mice per group. Hoechst (blue) staining reveals the cell layers in the retina. Scale bar 50μm. **(C)** Quantification of rod and cone outer segment thickness in the central retina in control vs. Norgestrel-fed rd10 mice. N = 3 mice per group, n = 8 sections per mouse. Results are presented as mean ± SEM (t-test, *p<0.05, ****p<0.0001).

In order to assess preservation of photoreceptor outer segments with Norgestrel diet, markers for rod (rhodopsin; red) and cone (PNA; green) outer segments were used on retinal sections. Outer segments were longer and denser in the central retina of Norgestrel treated mice compared to control (Fig 3A and 3B). This morphological change was observed from P15 for rods and from P20 for cones in the central retina (Fig 3A and 3B). Quantification of rod and cone outer segment length confirmed significant preservation out to P25 (Fig 3C).

### Microglial response in the rd10 retina is significantly altered with Norgestrel treatment

**Fig 4:**
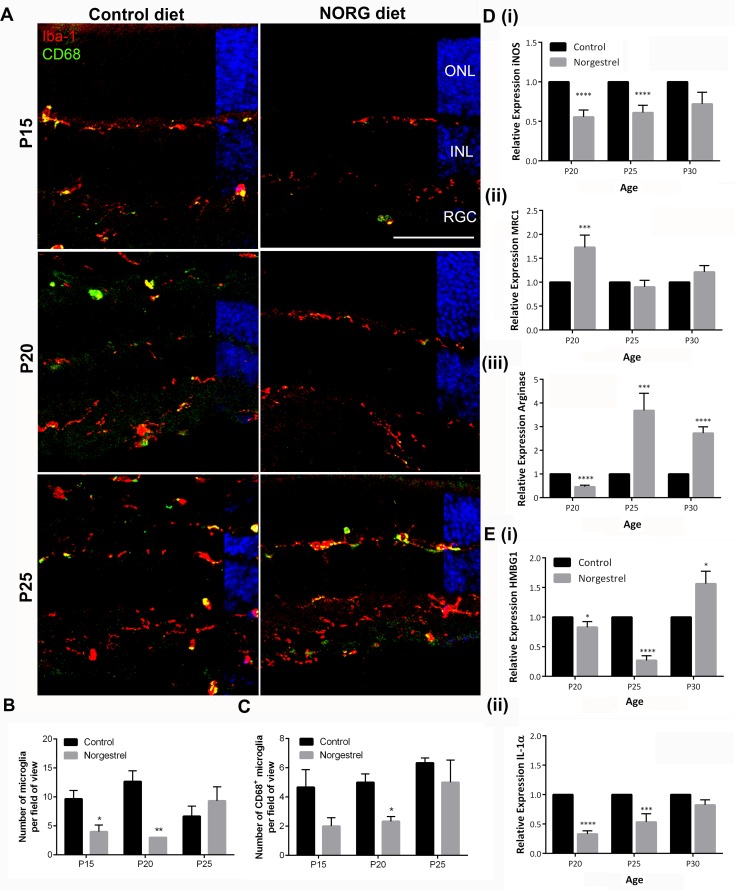
Norgestrel dampens pro-inflammatory microglial activity *in vivo* and decreases DAMP release from photoreceptors. **(A)** Confocal microscopic images of microglia (Iba1; red) and activated microglia (CD68; green) in the central retina from P15-P25 control and Norgestrel-fed rd10 mice. Less microglia including CD68+ microglia were observed at P15 and P20 in Norgestrel-fed mice. Hoechst (blue) staining reveals the cell layers in the retina. Scale bar 50μm. N = 3 mice per group, n = 8 sections per mouse. **(B & C)** Quantification of microglial number and CD68-positivity confirmed (B) significantly less microglia at P15 and P20 and (C) significantly less CD68-positivity at P20 with Norgestrel-supplementation. **(D)** RT-qPCR analysis of mRNA encoding (i) pro-inflammatory (M1) and (ii) & (iii) anti-inflammatory (M2) microglial markers in whole retina from P20-P30 control and Norgestrel-fed rd10 mice. Norgestrel significantly reduced pro-inflammatory and increased anti-inflammatory markers in the retina. Control column represents age-matched rd10 mice on control diet. N = 3 mice per group, n = 3 technical replicates per mouse. **(E)** RT-qPCR analysis of mRNA encoding danger associated molecular patterns (DAMP), (i) high motility box group 1 (HMGB1) and (ii) interleukin-1α (IL-1α) in whole retina from P20-P30 control and Norgestrel-fed rd10 mice. Norgestrel significantly reduced DAMP expression at P20 and P25 in Norgestrel-fed mice. Control column represents age-matched rd10 mice on control diet. N = 3 mice per group, n = 3 technical replicates per mouse. Results are presented as mean ± SEM (t-test, *p<0.05, ***p<0.005, ****p<0.0001).

Previous studies have highlighted a role for microglia in potentiating photoreceptor cell death in the rd10 mouse by driving inflammatory processes [18, 19]. We therefore sought to study microglial dynamics in rd10 mice following administration of Norgestrel. Amoeboid microglia (ionized calcium-binding adapter molecule 1; Iba1) were observed surrounding the ONL (Fig 4A), during the peak of cell death (P15 and P20) in the control retina as previously reported [9, 18]. Some stained positively for cluster of differentiation 68 (CD68), a marker used to identify phagocytic, activated microglia [30]. Conversely, microglia were ramified in morphology in Norgestrel-fed mice (P15, P20) and showed less CD68 immunoreactivity than control (Fig 4A). Norgestrel-diet significantly reduced microglial number at P15 and P20 (Fig 4B) and CD68-positivity at P20 (Fig 4C). The apparent correlation between significant photoreceptor protection and less microglial activation in Norgestrel-fed animals (Fig 4A–4C), implicates microglia in the cell death observed in rd10 mice. Inducible nitric oxide synthase (iNOS) is a marker used to identify M1 microglia in the rodent retina [31]. Markers of anti-inflammatory microglia (M2) have also been characterized, including mannose receptor C, type 1 (CD206/MRC1) [32] and arginase [30]. mRNA analysis on whole retina confirmed a Norgestrel-driven, significant decrease in iNOS expression (M1) at P20 and P25, and an increase in MRC1 (M2) at P20 and arginase (M2) expression at P25 and P30 (Fig 4D). High mobility group box 1 protein (HMGB1) and interleukin 1α (IL-1α) belong to the family of danger-associated molecular pattern molecules (DAMPs), expressed and released by damaged cells to trigger inflammation [33–36]. mRNA analysis of DAMP expression showed that Norgestrel induced a significant decrease in HMBG1 and IL-1α at P20 and P25 (Fig 4E), coinciding with increased photoreceptor survival (Fig 1), less M1 and increased M2 microglial activation (Fig 4A–4D).

### Norgestrel significantly reduces rd10 microglial-driven, cell contact-dependent degeneration of 661W cells

In order to evaluate the contribution of microglia to the course of photoreceptor cell loss in the rd10 retina in more detail, we utilized primary cell cultures of P16 rd10 retinal microglia. Culture purity was confirmed (S2 Fig). Due to the difficulty in isolating pure photoreceptor primary cultures and in order to assess the interaction between microglia and photoreceptors, we utilized the cone photoreceptor-like 661W cell line. Our group has previously used 661Ws as an *in vitro* model to study how photoreceptors respond to stressful stimuli and to understand how Norgestrel works in providing neuroprotection [11–13].

**Fig 5:**
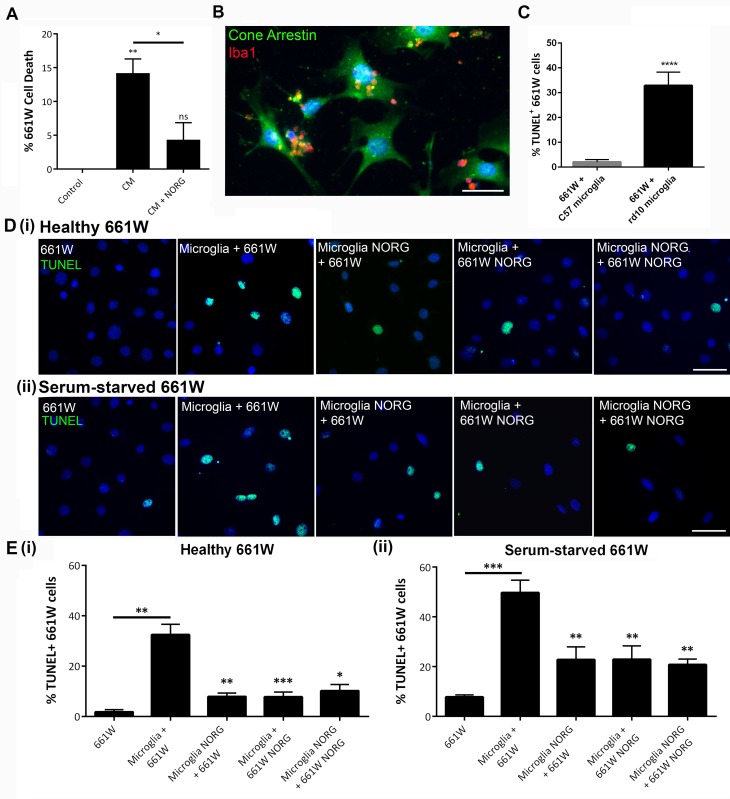
Norgestrel significantly abrogates rd10 microglial-induced 661W cell death. **(A)** Conditioned media was collected from primary microglial cells cultured with (CM + NORG), or without (CM) 20 μM Norgestrel over 24 h. 661W cells were cultured over a further 24h in the absence (Control) or presence of conditioned media (CM, CM + NORG) and percentage cell death was assessed by the MTS assay. Exposing 661Ws to CM from rd10 microglia resulted in increased neuronal cell death whereas pre-treatment of rd10 microglia with Norgestrel significantly reduced cell death. N = 6 biological replicates, n = 4 technical replicates per group. (One way ANOVA followed by Tukey’s post-hoc test,). **(B)** Representative fluorescent microscopic image of primary rd10 microglial cells (Iba1; red) in culture with 661W cells (Cone arrestin; green). Hoechst (blue) staining reveals cell nuclei. Scale bar 30μm. **(C)** 661W cells were cultured in the presence of C57 or rd10 primary retinal microglia. Apoptosis of 661W cells as detected by TUNEL of DNA strand breaks in cell nuclei revealed significantly more 661W cell death when cultured with rd10 microglia. **(D)** 661W cells were cultured in (i) the presence of (healthy 661W) or (ii) absence of (serum-starved 661W) serum over 3 h. These cells were simultaneously treated with either 20 μM Norgestrel (661W NORG) or vehicle control (661W). The treated 661W cells were then placed in culture with microglia that had been treated with (Microglia NORG) or without (Microglia) 20 μM Norgestrel over 24h. Co-cultures were incubated for a further 24h. Apoptosis of 661W cells was detected by TUNEL of DNA strand breaks in cell nuclei. All Norgestrel-treated co-cultures had significantly less TUNEL-positive 661W cells than equivalent vehicle controls. Hoechst (blue) staining reveals the cell nuclei. Scale bars 50μm. **(E)** Quantification of TUNEL-positive 661W cells cultured in (i) the presence of (healthy 661W) or (ii) the absence of (serum-starved 661W) serum and following Norgestrel or vehicle DMSO treatment and co-incubation with primary rd10 microglial cells pre-treated with Norgestrel or vehicle DMSO. N = 8 mice for primary culture, n = 6 technical replicates per group. Results are presented as mean ± SEM (t-test, *p<0.05, **p<0.01, ***p<0.005, ****p<0.0001).

To determine the effects of released factors from rd10 microglia on 661W viability, healthy 661Ws were cultured for 24 h with conditioned medium (CM) from rd10 microglia pre-treated with 20 μM Norgestrel or equivalent DMSO control. 661W cell viability was then assessed using the MTS assay. Whilst CM from DMSO-treated microglia resulted in a ~14% increase in 661W cell death, CM from Norgestrel-treated microglia resulted in only a ~4% increase compared to untreated control (Fig 5A). This suggests a novel role for Norgestrel in modulating microglial responses in the rd10 retina.

We hypothesized that direct cell-cell contact may regulate microglial responses [37, 38], for in the early stages of rd10 retinal degeneration, microglia associate closely with cells in the ONL [9, 18]. Therefore, a co-culture assay was designed using a combination of rd10 microglia, healthy 661Ws and serum-starved 661Ws. This enabled us to analyze the direct effects of Norgestrel on microglia and photoreceptors in a way that more closely resembled the *in vivo* state. Prior to co-culture, primary rd10 microglia and 661W cells were treated with 20 μM Norgestrel or equivalent DMSO control (24 h microglia, 3 h for 661Ws). 661W cells were then added to the microglia and incubated for a further 24 h. During this time, microglia appeared to migrate and settle in clusters close to 661W cells (Fig 5B). Subsequent cell viability of co-cultured 661W cells was assessed using the TUNEL assay.

After 24h of co-culture, ~32% of healthy 661W cells cultured with microglia were TUNEL positive compared to ~1.8% of 661W cells cultured in isolation (Fig 5D (i) and 5E (i)). This strongly suggests that direct rd10 microglia-661W cell contact will result in higher levels of cell death, than when 661W cells are exposed to secreted microglial factors alone (compare Fig 5A to 5E (i)). Retinal microglia from age-matched C57 mice treated identically to rd10 microglia resulted in 2.1% 661W cell death (Fig 5C), confirming that rd10 microglia specifically drive neuronal cell death.

The previous experiment was replicated using serum-starved 661W cells, a previously established method for inducing 661W cellular stress [11, 13]. This allowed us to investigate the response of microglia to damaged photoreceptors, in an attempt to mimic photoreceptor stress present in the rd10 mouse retina. 661Ws were serum-starved for 3 h, resulting in ~8% cell death as assessed by TUNEL (Fig 5E (ii)). Addition of rd10 primary microglial cells for 24 h increased 661W cell death to ~50%, a 42% increase in cell death compared to serum-starved 661W cells cultured in isolation (Fig 5D (ii) and 5E (ii)).

Treatment of rd10 microglia with 20 μM Norgestrel prior to co-culture significantly reduced their ability to damage healthy and serum-starved 661W cells (Fig 5E (i) (ii)), indicating that Norgestrel can work directly on microglia. Interestingly, pre-treatment of 661W cells with 20 μM Norgestrel also resulted in increased 661W protection (Fig 5E (i) (ii)), demonstrating Norgestrel’s ability to prime undamaged and stressed photoreceptors against microglial-derived harmful stimuli.

### Norgestrel modulates pro- and anti-inflammatory states in rd10 microglia

**Fig 6:**
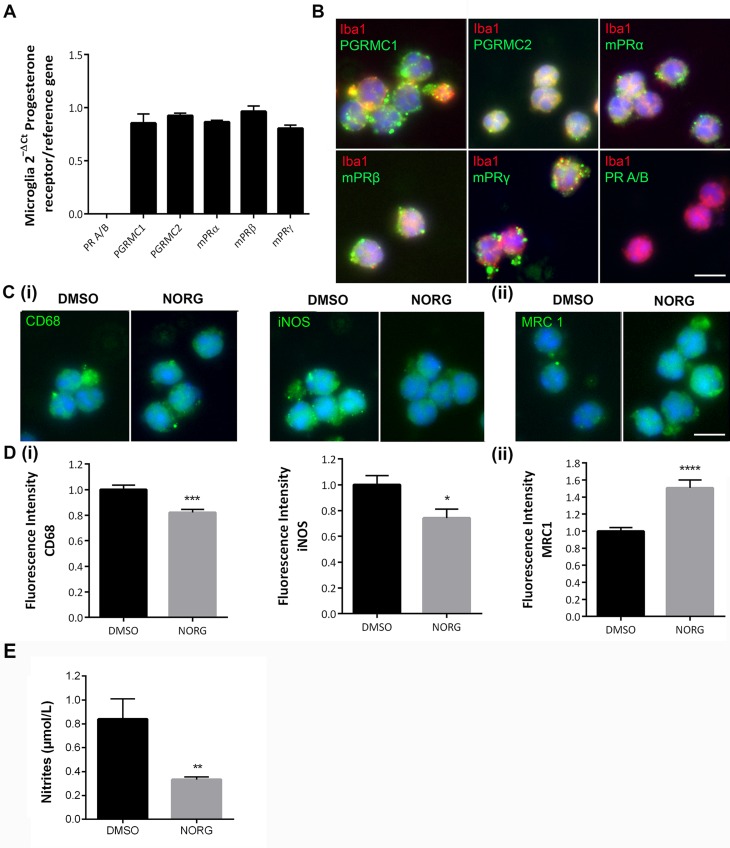
Norgestrel reduces pro-inflammatory and promotes anti-inflammatory phenotypes in rd10 microglia. **(A)** RT-qPCR analysis detected similar levels of mRNAs encoding progesterone receptor membrane complexes 1 and 2 (PGRMC1, PGRMC2) and all three membrane progesterone receptor isoforms, α, β and γ (mPRα, mPRβ, mPRγ) in rd10 retinal microglial cells. Classical progesterone receptor (PR A/B) was not expressed. Actin, HPRT and GAPDH served as reference genes. **(B)** Immunolabeling confirmed the presence of these progesterone receptors in primary rd10 microglial cells. Hoechst (blue) staining reveals the cell nuclei. Scale bar represents 10μm. **(C)** Fluorescent microscopic images of primary rd10 microglia immunolabeled for (C) (i) markers of activation (CD68) and pro-inflammation (M1) (iNOS) and (C) (ii) anti-inflammatory activation (M2) (MRC1); after treatment with 20 μM Norgestrel over 24h. Norgestrel reduced pro-inflammatory and increased anti-inflammatory markers in rd10 microglia. Scale bar 10μm. **(D)** Quantification of fluorescence intensity of (D) (i) CD68 and iNOS and (D) (ii) MRC1 in primary rd10 microglial cultures following Norgestrel treatment over 24h. N = 8 mice, n = 4 technical replicates per group. **(E)** Nitrite concentration in the media of rd10 microglia *in vitro* treated with Norgestrel or vehicle over 24hr. Norgestrel significantly reduced nitrite release from rd10 microglia. N = 8 biological replicates, n = 2 technical replicates per sample. Results are presented as mean ± SEM (t-test, *p<0.05, ***p<0.005, ****p<0.001).

Norgestrel can act directly on rd10 microglia and consequently prevent microglial-driven 661W cell death (Fig 5). Norgestrel mediates its neuroprotective effects through progesterone receptors [11]. mRNA analysis revealed similar expression patterns of progesterone receptor membrane complexes 1 and 2 (PGRMC1, PGRMC2), and membrane progesterone receptors α, β and γ (mPRα, β, γ) in rd10 microglia (Fig 6A). Immunofluorescence confirmed expression of these receptors (Fig 6B). Classical progesterone receptors A and B (PRA/B) were not detected in these cells (Fig 6A and 6B). Primary rd10 microglia were subsequently treated with 20 μM Norgestrel and analyzed by immunofluorescence for M1 and M2 state markers. Norgestrel induced significantly lower levels of CD68 and iNOS (M1) and higher levels of MRC1 (M2) in rd10 microglia compared to control (Fig 6C). Nitric oxide production was assessed indirectly by measuring endogenous nitrite levels in media from primary rd10 microglia treated with Norgestrel or vehicle. Norgestrel significantly lessened endogenous microglial nitrites (Fig 6E). Thus, we hypothesize that Norgestrel dampens pro-inflammatory activity and stimulates anti-inflammatory states in rd10 microglia, resulting in increased protection of photoreceptors.

### Norgestrel acts as an anti-inflammatory in stressed 661W cells and in rd10 mice

**Fig 7:**
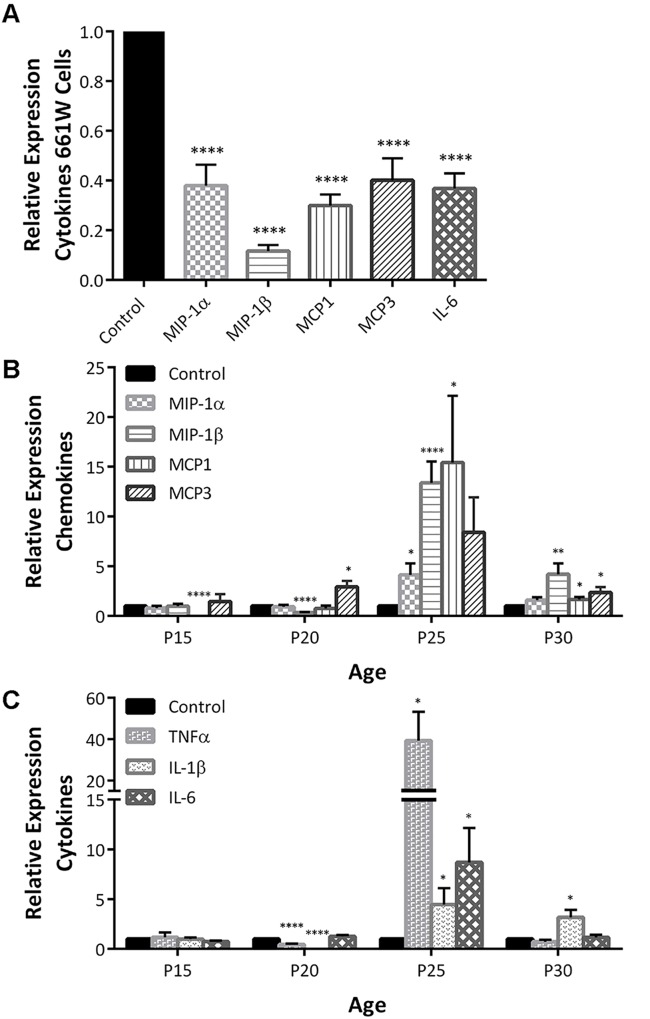
Norgestrel reduces inflammation in 661Ws and the rd10 retina at P15 and P20. **(A)** 661W cells were serum-starved and treated with 20μM Norgestrel or the equivalent DMSO control (Control) over 24h. RT-qPCR analysis detected a significant decrease in mRNA levels of macrophage inflammatory protein 1α and 1β (MIP-1α, MIP-1β), monocyte chemoattractant proteins 1 and 3 (MCP1, MCP3) and interleukin 6 (IL-6) in serum-starved cells treated with Norgestrel. N = 6 biological replicates, n = 3 technical replicates per sample. **(B-C)** Retinal cytokine mRNA levels were measured by RT-qPCR analysis, in P15-30 rd10 mice. Relative (B) chemokine (MIP-1α, MIP-1β, MCP1 and MCP3) and (C) pro-inflammatory cytokine (tumour necrosis factor α (TNFα), interleukin 1β (IL-1β) and interleukin 6 (IL-6)) mRNA expression levels in retinas of rd10 mice supplemented with Norgestrel. Control column represents age-matched rd10 mice on control diet. N = 3 mice per group, n = 3 technical replicates per mouse. Results are presented as mean ± SEM (t-test, *p<0.05, **p<0.01, ****p<0.001).

Microglial infiltration and phenotypic activation is driven by differential chemokine signaling [39, 40]. Several cytokines and chemokines including MIP-1α (CCL3), MIP-1β (CCL4), MCP-1 (CCL2), MCP-3 (CCL7), TNF-α, IL-1β and IL-6 have been implicated in photoreceptor-microglial crosstalk and retinal degeneration [18, 31, 41–45]. Thus we sought to elucidate how Norgestrel treatment affected expression of these cytokines and chemokines. Both in stressed 661W cells (Fig 7A) and during the early stages (P15 –P20) of rd10 disease progression (Fig 7B), Norgestrel successfully acts as an anti-inflammatory agent, downregulating cytokine gene expression. This downregulation is concurrent with reduced gene transcription of pro-inflammatory mediators TNFα, IL-1β and IL-6 (Fig 7C). Secreted from retinal microglia, these cytokines likely act on photoreceptors to potentiate cell death [18, 46–48]. A loss of photoreceptors at P25 correlates with an upregulation of chemokine and cytokine gene expression (Fig 7B and 7C) and infiltration of microglia to the ONL (Fig 4A).

### Norgestrel upregulates neuroprotective fractalkine-CX3CR1 signaling in the rd10 retina

**Fig 8:**
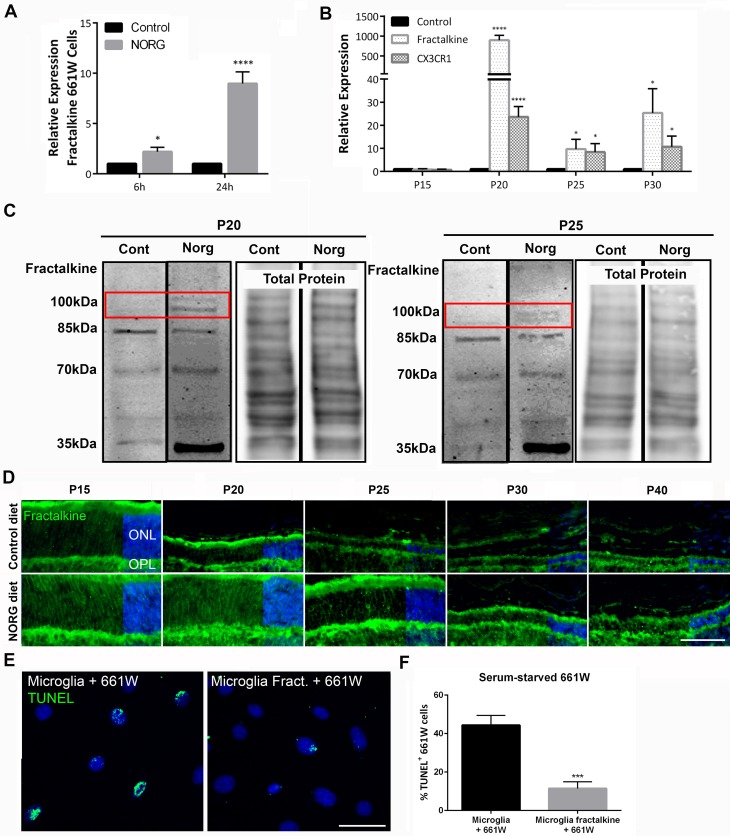
Norgestrel upregulates fractalkine-CX3CR1 signaling in the rd10 retina. **(A)** 661W cells were serum-starved and treated with 20 μM Norgestrel (Norgestrel) or the equivalent DMSO control (Control) over 6 and 24h. RT-qPCR analysis detected a significant increase in mRNA levels of fractalkine in serum-starved cells treated with Norgestrel. N = 6 biological replicates, n = 3 technical replicates per sample. **(B)** Norgestrel-supplementation in rd10 mice induced a significant increase in fractalkine and CX3CR1 mRNA expression in P20-P30 retinas compared to control. Control column represents age-matched rd10 mice on control diet. N = 3 mice per group, n = 3 technical replicates per mouse. **(C)** Western blot for fractalkine at P20 and P25 confirmed an increase in full-length fractalkine (100kDa; red box) at both time-points with Norgestrel treatment. Total protein stain shows similar loading between samples. Blots are representative of 6 biological replicates per group. **(D)** Fluorescent microscopic images of fractalkine (green) in the central retina from P15-40 of control and Norgestrel-fed rd10 mice. Fractalkine staining is evident in the outer nuclear layer (ONL) of the retina in Norgestrel-supplemented mice. Hoechst (blue) staining reveals the cell layers in the retina. Scale bar 50μm. N = 3 mice per group, n = 3 sections per mouse. **(E)** 661W cells were cultured in the absence of serum (serum-starved 661W) over 3 h. 661W cells were then placed in culture with microglia that had been treated with (Microglia Fract.) or without (Microglia) 1.2μg/ml recombinant full-length fractalkine over 24h. Co-cultures were incubated for a further 24h. Apoptosis of 661W cells was detected by TUNEL of DNA strand breaks in cell nuclei. Co-cultures with fractalkine-treated microglia had less TUNEL-positive 661W cells than equivalent vehicle control. Scale bar 30μm. **(F)** Quantification of TUNEL-positive 661W cells cultured in the absence of serum and following co-incubation with primary rd10 microglial cells pre-treated with recombinant full-length fractalkine or vehicle. N = 9 mice for primary culture, n = 5 technical replicates per group. Results are presented as mean ± SEM (t-test, *p<0.05, ***p<0.005, ****p<0.001).

Fractalkine (CX3CL1) is a chemokine synthesized as a 50-75kDa protein [49]. It is glycosylated forming a 100kDa isoform, which is membrane-bound [49–51]. Full-length fractalkine consists of a chemokine domain with CX3C motif, highly-glycosylated mucin-like stalk, a transmembrane domain and a short cytoplasmic domain [52]. Membrane-bound fractalkine is cleaved by metalloproteinases, such as ADAM10, to release soluble fractalkine (85kDa) [53]. Fractalkine is also recycled from the membrane and stored in intracellular vesicles [51, 54], possibly as a 95kDa isoform [50]. Signaling to its receptor CX3CR1 on microglia [55], both *in vivo* and *in vitro* studies have shown that complementary fractalkine-CX3CR1 signaling can modulate microglial activation and migration resulting in improved neuronal survival [56–61]. Therefore, we explored Norgestrel’s ability to upregulate fractalkine and CX3CR1 gene expression, in the degenerating rd10 retina. Norgestrel treatment of stressed 661W cells significantly upregulated fractalkine mRNA over 24 h (Fig 8A). In the rd10 retina, Norgestrel significantly increased fractalkine levels at P20 by over 1000-fold that of the control (Fig 8B); coinciding with significant protection of the ONL (Fig 1). Norgestrel also significantly upregulated CX3CR1 gene expression (Fig 8B). Norgestrel-induced fractalkine signaling was dampened at later time points (P25, P30) compared to P20 (Fig 8B) correlating with photoreceptor loss in Norgestrel-treated mice (Fig 1). Western blotting revealed an increase in 100kDa fractalkine protein at P20 and P25 with Norgestrel (Fig 8C; red boxes). This isoform is neuroprotective in the retina by dampening harmful microglial processes and consequently improving photoreceptor survival [57, 62]. Immunofluorescence on retinal sections also showed an increase in fractalkine with Norgestrel (Fig 8D). Fractalkine was found predominantly in the ONL and outer plexiform layer (OPL), and staining was increased in all (P15-P40) Norgestrel-fed mice compared to control (Fig 8D). Primary rd10 microglia treated with recombinant full-length fractalkine for 24 h and subsequently co-cultured with serum-starved 661W cells induced significantly less 661W cell death compared to vehicle (Fig 8E and 8F). This suggests that Norgestrel drives an upregulation of fractalkine in the retina, which will reduce microglial-driven neuronal cell death.

## Discussion

In this study we demonstrate the protective properties of the synthetic progestin ‘Norgestrel’, when administered *via* non-invasive diet supplementation. Previously shown to protect through an upregulation of growth factors [12, 13], this study highlights a novel role for Norgestrel, in acting directly upon microglial cells to dampen damaging inflammatory processes. This is an important finding as recent literature has shown that microglial cells may potentiate retinal disease [18, 19, 21, 44, 63, 64]. We also provide further mechanistic insight, showing that Norgestrel drives up fractalkine expression in the rd10 retina, which likely acts to further dampen microglial activation.

Our group has previously shown that Norgestrel is neuroprotective in the rd10 retina when administered *via* intraperitoneal injection from P18 [10]. Since this study, we have demonstrated that significant morphological changes take place in the rd10 retina prior to photoreceptor cell loss [9]. In light of this finding, we sought to administer Norgestrel from an earlier time-point. We also wished to use a less invasive method and avoid the use of the vehicle DMSO, which has been shown to significantly affect cell metabolism in several ways including acting as an anti-inflammatory [65]. Using a supplemented-diet from P10, we have demonstrated increased protection with Norgestrel in the rd10 retina (Fig 1) compared to intra-peritoneal injection from P18 [10].

We hypothesized that rd10 retinal microglia drive targeted neuronal degeneration of viable cells in a cell autonomous manner. In order to substantiate this claim, and subsequently investigate the direct effects of Norgestrel on microglia, we utilized *in vitro* cultures of 661W cone photoreceptor-like cells and primary rd10 microglia both in isolation and in co-culture. By culturing 661Ws with Norgestrel-treated rd10 microglial conditioned media, we show that Norgestrel dampens damaging inflammatory factor secretion (Fig 5A). Interestingly, direct microglia-photoreceptor contact induces significantly more cell death of healthy 661Ws than conditioned media alone (compare Fig 5A to 5E (i)). Extrapolating this to the *in vivo* situation, this suggests that microglia can cause higher levels of cell death when they have reached the ONL and are in direct contact with photoreceptors. These results also confirm our hypothesis, showing that isolated rd10 microglia drive targeted cell death of healthy photoreceptors, as primary C57 microglia did not induce 661W cell death. Inducing ~30% and ~42% cell death in healthy and serum-starved 661W cells respectively, 661W cells are more vulnerable to damaging microglial-contact when already stressed (compare Fig 5D (i) to 5D (ii)). Importantly, Norgestrel alleviates microglial-driven neuronal death of healthy and stressed 661Ws, by acting directly on both microglia and 661W cells. Of course, microglial-driven cell death is initiated due to effects from the mutation in *pde6b*. Microglia are therefore stimulated to adopt a damaging phenotype by signals from other cell types in the retina. However, once activated microglia will likely promote further degeneration of viable photoreceptors, exacerbating disease progression.

We suggest that Norgestrel works through progesterone receptors present on microglial cells to reduce pro-inflammatory M1 and stimulate anti-inflammatory M2 phenotypes (Figs 4 & 6). Ultimately, these actions preserve photoreceptor cell viability. This finding was supported by rt-qPCR studies on whole retinas (Fig 7), whereby Norgestrel lead to a significant decrease in pro-inflammatory TNFα and IL-1β gene expression, both expressed by retinal microglia [66].

Norgestrel-diet administration prevented any photoreceptor loss between P15 and P20 in rd10 mice (Fig 1 & S1 Fig). We believe this protection is largely owed to increased levels of Norgestrel-induced fractalkine signaling. Norgestrel upregulated fractalkine expression by over 1000-times that of P20 control (Fig 8). Fractalkine signaling has been shown to prevent cell loss in a number of different disease models [56, 61, 67–70]. In mouse models of RP, CX3CR1^-/-^ rd10 mice experience a worsened disease progression [19] and intravitreal injection of recombinant full-length fractalkine improves photoreceptor survival in the rd10 mouse [62]. Indeed we show that rd10 microglia pre-treated with full-length fractalkine cause significantly less 661W cell death in co-culture (Fig 8E and 8F). These studies stand to corroborate the crucial importance of fractalkine-CX3CR1 signaling in retinal neuroprotection and the potential of Norgestrel as a therapeutic for RP.

Of particular interest, full-length fractalkine appears to play a neuroprotective role by dampening harmful microglial activity [57, 62], whereas soluble fractalkine may potentiate disease progression by increasing microglial migration [57]. In support of this, fractalkine cleavage increases prior to neuronal cell death *in vitro* [71] and loss of membrane-bound fractalkine precedes degeneration in the rd10 retina [50]. Western blots of P20 retinas revealed an increase in full-length (Fig 8C; 100kDa) and a decrease in soluble (Fig 8C; 85kDa) isoforms of fractalkine with Norgestrel.

Ultimately however, Norgestrel’s neuroprotective effects are gradually lost from P25, though Norgestrel-driven photoreceptor survival does not lapse entirely until P50 (Fig 1). It is worth noting that the measurable neuroprotective effects observed with Norgestrel are in line with previous studies documenting other types of neuroprotective strategies in the rd10 retina [18, 19, 62]. Such studies similarly present therapeutic approaches resulting in delayed degeneration rather than complete prevention of photoreceptor loss. This is likely due to the fact that the primary insult remains, the mutation in *pde6b*, and will therefore persist as a source of cellular stress. Norgestrel-treated mice show a significant loss in fractalkine at the RNA and protein level at P25 compared to P20 (Fig 8B–8D). Taken together with the bulk of evidence supporting fractalkine’s neuroprotective properties in the retina, we believe that loss of fractalkine signaling is detrimental to photoreceptors. We hypothesize that such a loss in fractalkine signaling at P25 contributes to microglial infiltration to the ONL (Fig 4A) and subsequent potentiation of cell death (Fig 5).

In conclusion, this study outlines novel and desirable properties of the synthetic progestin ‘Norgestrel’, in acting directly on retinal microglia, and indirectly through photoreceptors, to dampen damaging microglial activation *in vitro* and *in vivo*. The data presented suggest a critical role for Norgestrel-induced fractalkine signaling in the dampening of microglial activation. Future studies will be aimed at further elucidating the role that fractalkine signaling plays in retinal degeneration and Norgestrel-associated neuroprotection. We believe direct photoreceptor neuroprotection should remain at the forefront of RP immunotherapy development: targeting mutated photoreceptors would alleviate subsequent photoreceptor-microglial cell crosstalk, minimizing consequential microglial-driven photoreceptor cell loss. Additional anti-inflammatory properties may then further prevent microglial responses shown to exacerbate disease progression.

## Supporting Information

S1 Fig Representative images of retinal morphology along the vertical meridian of the eye of C57, rd10 control diet (rd10) and rd10 Norg diet (rd10 Norg) at P20. Retinal sections were labelled with the nuclear stain, Hoechst, to demonstrate the loss of photoreceptors in the rd10 retina (C57 vs rd10) and the neuroprotective effects of Norgestrel (rd10 vs rd10 Norg). White boxes illustrate the regions used to image the central and peripheral retina. Scale bar 300μm.
(TIF)

S2 Fig Characterization of a pure microglial culture. **(A & B)** Fluorescent microscopic images of primary rd10 microglial cells *in vitro* confirmed a pure microglial cell population. Hoechst (blue) staining reveals the cell nuclei. **(A)** Antibodies targeted against astrocytes (GFAP; red), Müller glial cells (glutamate synthase (GS); green), retinal ganglion cells (RGCs) (neurofilament medium (NFM); green), amacrine cells (syntaxin 1; red), horizontal cells (calbindin; red), bipolar cells (CEH10 homeodomain-containing homolog (CHX10); green) and rods (rhodopsin (Rhod); red) showed no positive staining within the isolated population of cells. Small quantities of non-nuclear (Hoechst negative) debris from these cells was found in culture (see panel inserts). Scale bar 10μm. **(B)** Confocal microscopic XY image of primary rd10 microglial cells *in vitro* (Iba1; red). Secondary only control (panel insert) confirmed specific staining of the antibody. Scale bar 10μm.
(TIF)
