# Supplementary figures and images for: Progesterone Attenuates Microglial-Driven Retinal Degeneration and Stimulates Protective Fractalkine-CX3CR1 Signaling
Source: PLoS One. 2016 Nov 4;11(11):e0165197. doi: 10.1371/journal.pone.0165197 (PMC5096718; doi:10.1371/journal.pone.0165197)

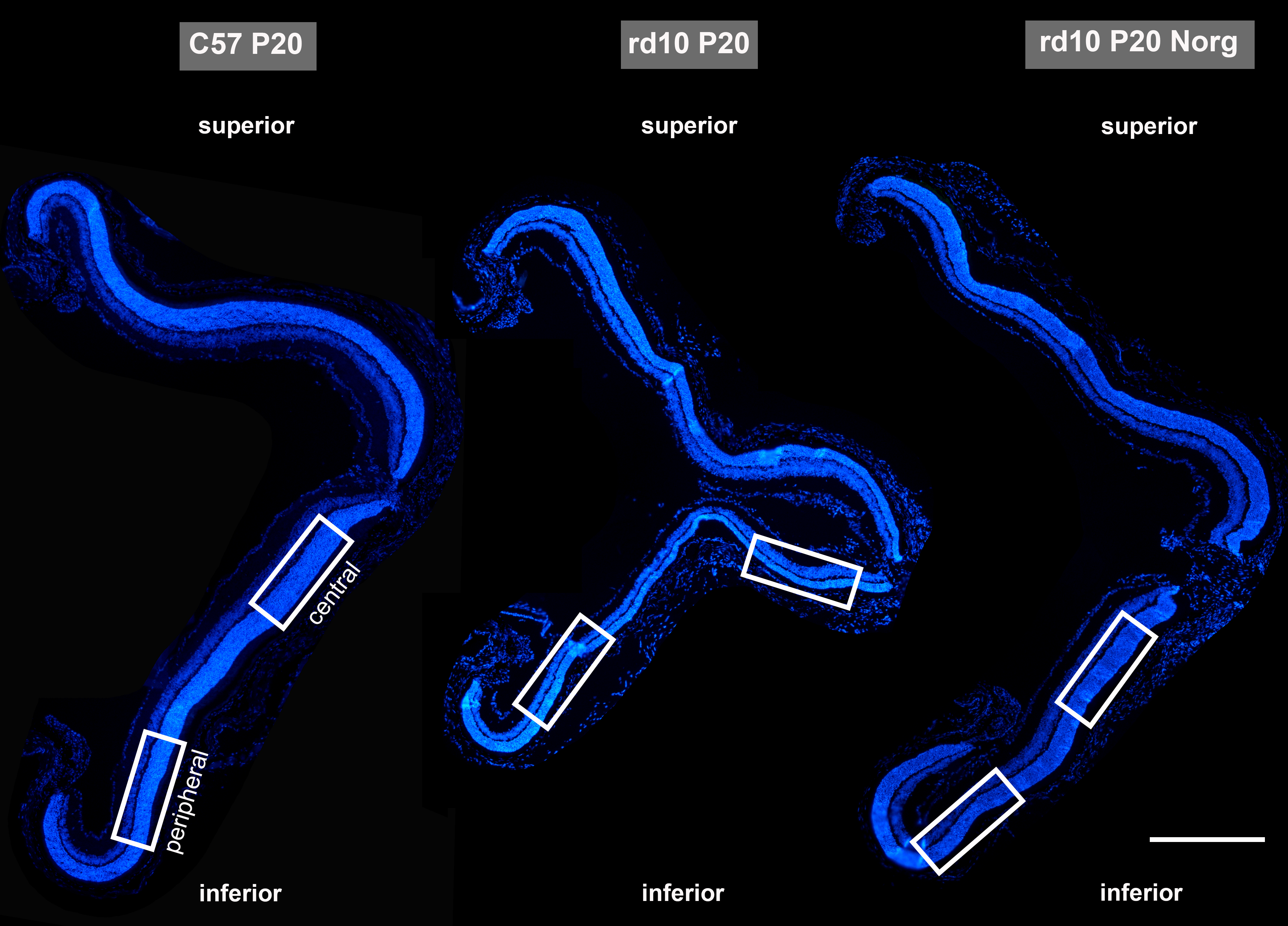

Supplement: S1 Fig — Retinal sections were labelled with the nuclear stain, Hoechst, to demonstrate the loss of photoreceptors in the rd10 retina (C57 vs rd10) and the neuroprotective effects of Norgestrel (rd10 vs rd10 Norg). White boxes illustrate the regions used to image the central and peripheral retina. Scale bar 300μm. (TIF) [file pone.0165197.s001.tif]

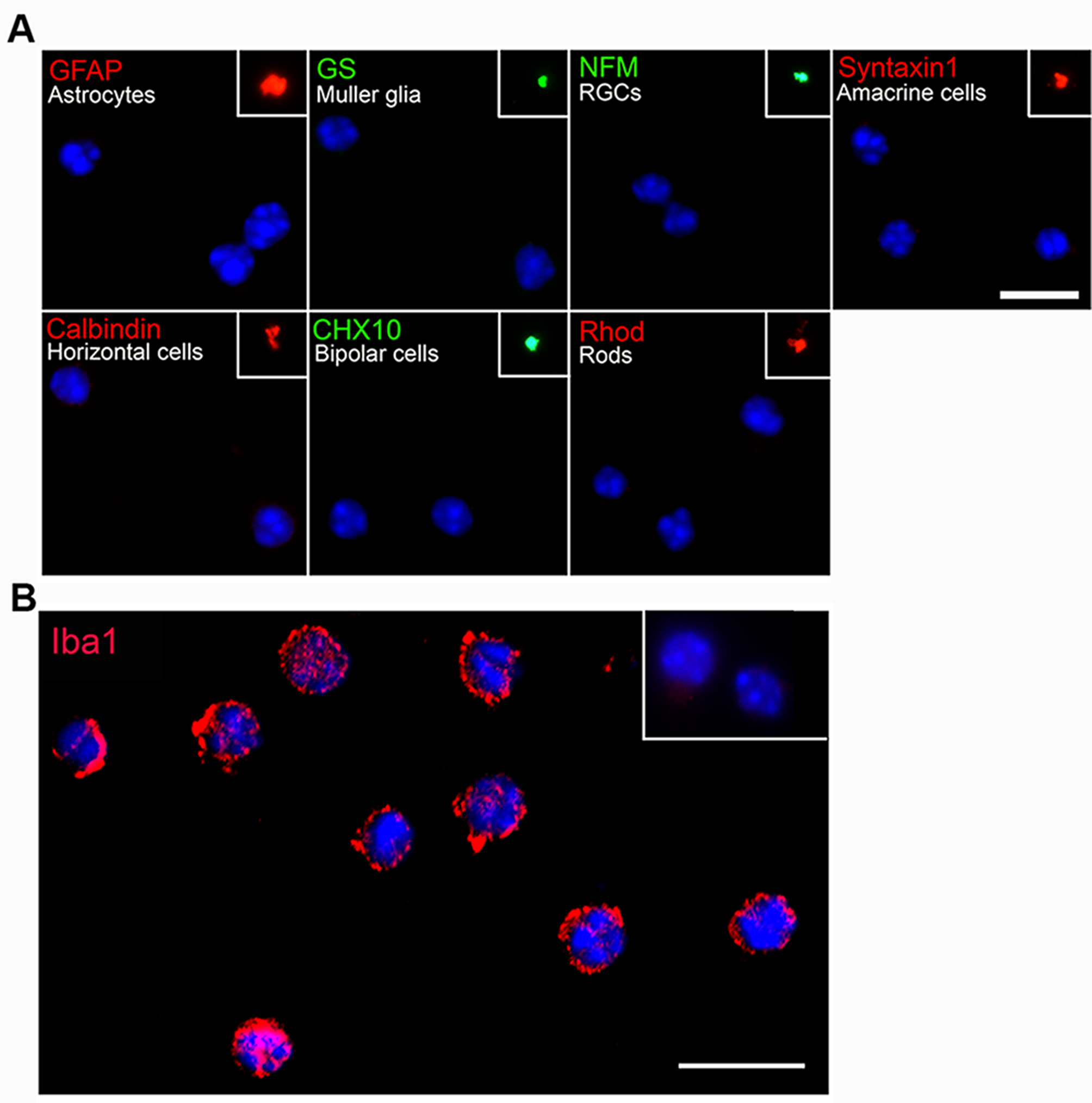

Supplement: S2 Fig — (A & B) Fluorescent microscopic images of primary rd10 microglial cells in vitro confirmed a pure microglial cell population. Hoechst (blue) staining reveals the cell nuclei. (A) Antibodies targeted against astrocytes (GFAP; red), Müller glial cells (glutamate synthase (GS); green), retinal ganglion cells (RGCs) (neurofilament medium (NFM); green), amacrine cells (syntaxin 1; red), horizontal cells (calbindin; red), bipolar cells (CEH10 homeodomain-containing homolog (CHX10); green) and rods (rhodopsin (Rhod); red) showed no positive staining within the isolated population of cells. Small quantities of non-nuclear (Hoechst negative) debris from these cells was found in culture (see panel inserts). Scale bar 10μm. (B) Confocal microscopic XY image of primary rd10 microglial cells in vitro (Iba1; red). Secondary only control (panel insert) confirmed specific staining of the antibody. Scale bar 10μm. (TIF) [file pone.0165197.s002.tif]
